# Mixed-methods process evaluation of the “Karl-Heinz” cardiac prehabilitation program in older patients: protocol for the PRECOVERY trial

**DOI:** 10.1186/s13063-026-09608-4

**Published:** 2026-03-18

**Authors:** Annemieke Munderloh, Carolin Steinmetz, Stephanie Heinemann, Christine A. F. von Arnim, Thomas Asendorf, Tim Mathes, Monika Sadlonova, Uta Sekanina, Eva Hummers, Christiane A. Müller

**Affiliations:** 1 Department of General Practice, University Medical Center Göttingen, Göttingen, Germany; 2 Department of Geriatrics, University Medical Center Göttingen, Göttingen, Germany; 3 German Center for Cardiovascular Research (DZHK), Partner Site Lower Saxony, Göttingen, Germany; 4 Department of Medical Statistics, University Medical Center Göttingen, Göttingen, Germany; 5 Department of Cardiothoracic and Vascular Surgery, University Medical Center Göttingen, Göttingen, Germany; 6 Department of Psychosomatic Medicine and Psychotherapy, University Medical Center Göttingen, Göttingen, Germany

**Keywords:** Process evaluation, Mixed-methods, Randomized controlled trial, Complex intervention, Prehabilitation, Older patients, Cardiac procedure, Structured qualitative content analysis

## Abstract

**Background:**

Structured cardiac prehabilitation programs for older patients prior to cardiac procedures address an unmet medical need, yet implementation evidence remains scarce. “Karl-Heinz” (*K*ognitiv & k*a*rdiale P*r*ehabi*l*itation vor *He*r*zin*terventionen; cognitive and cardiac prehabilitation prior to cardiac procedures) is a tailored prehabilitation program designed to improve quality of life and reduce mortality 12 months after elective cardiac procedures. PRECOVERY is a randomized, controlled, two-arm parallel group, assessor-blinded, multicenter trial evaluating its clinical efficacy. This study protocol outlines the embedded mixed-methods process evaluation examining both the delivery and implementation of “Karl-Heinz.” The objectives are (1) to examine the implementation process across prehabilitation centers, including contextual barriers and facilitators; (2) to assess the intervention’s fidelity, dose, adaptations, reach, and the quality of health professional training; and (3) to analyze key stakeholders’ perspectives on implementation and perceived impact.

**Methods:**

The quantitative strand will collect standardized questionnaires from trial participants (*n* = 422, aged ≥ 65 years), their relatives, and health professionals from participating prehabilitation centers. Additional data sources include therapy plans and patient diaries. Furthermore, we will systematically analyze evaluation data from health professionals delivering the intervention to assess training delivery. The data will be analyzed using the “joint frailty model for longitudinal data and terminal event”, supplemented by exploratory analyses of main trial data.

The qualitative strand will include semi-structured interviews with a subsample of patients (*n* = 24) and relatives (*n* = 24) before and after the cardiac procedure. We will interview physicians performing the cardiac procedure (*n* = 9) and conduct focus groups (three with health professionals involved in prehabilitation delivery, two with general practitioners of prehabilitated patients). Qualitative data will be analyzed using structured qualitative content analysis. The results from both strands will be triangulated and related to the results from the main trial.

**Discussion:**

This process evaluation will provide detailed insights into the implementation and perceived impact of “Karl-Heinz.” Capturing perspectives from multiple stakeholder groups along with a detailed analysis of training processes will help to identify contextual factors, facilitators, barriers, and deviations. The mixed-methods approach will enable a thorough understanding, support the intervention’s refinement, optimize allocation of resources, overall reproducibility, and the assessment of possible integration into routine care.

**Trial registration:**

German Clinical Trials Register (DRKS; http://www.drks.de; DRKS00030526). Registered on 30 January 2023.

**Supplementary Information:**

The online version contains supplementary material available at 10.1186/s13063-026-09608-4.

## Introduction

### Background 

#### Clinical background and rationale for prehabilitation

Cardiovascular diseases (e.g., coronary heart disease and heart valve disease) pose threats to the well-being and functional capacity of older patients and remain the leading cause of death worldwide [1]. The prevalence of cardiovascular diseases is expected to rise due to demographic shifts [2]. While advancements in cardiac procedures have improved survival, older patients, especially those who are frail or multimorbid, face higher risks of peri- and postoperative complications [3–5]. A key contributing factor to these complications is reduced physiological reserve, including loss of muscle mass, strength, and endurance [3, 4]. Reduced physical function impairs quality of life (QoL), autonomy, as well as resilience, and prolongs recovery [6]. One central postoperative complication is postoperative delirium, which affects 12 to 53% of patients and correlates with poor long-term outcomes [7]. Frail and delirious patients experience an increased mortality rate of up to 40% [8, 9].

Prehabilitation aims to enhance a patient’s functional status and to optimize cognitive and psychological capacity prior to major procedures. It has emerged as a promising approach to target potential individual preprocedural risk factors and optimize the overall health status of elderly patients with heart disease [10–13]. While postprocedural rehabilitation for older and multimorbid patients has shown efficacy, structured preprocedural interventions like cardiac prehabilitation programs for this population are lacking [14].

#### PRECOVERY trial and the “Karl-Heinz” intervention

**Fig. 1:**
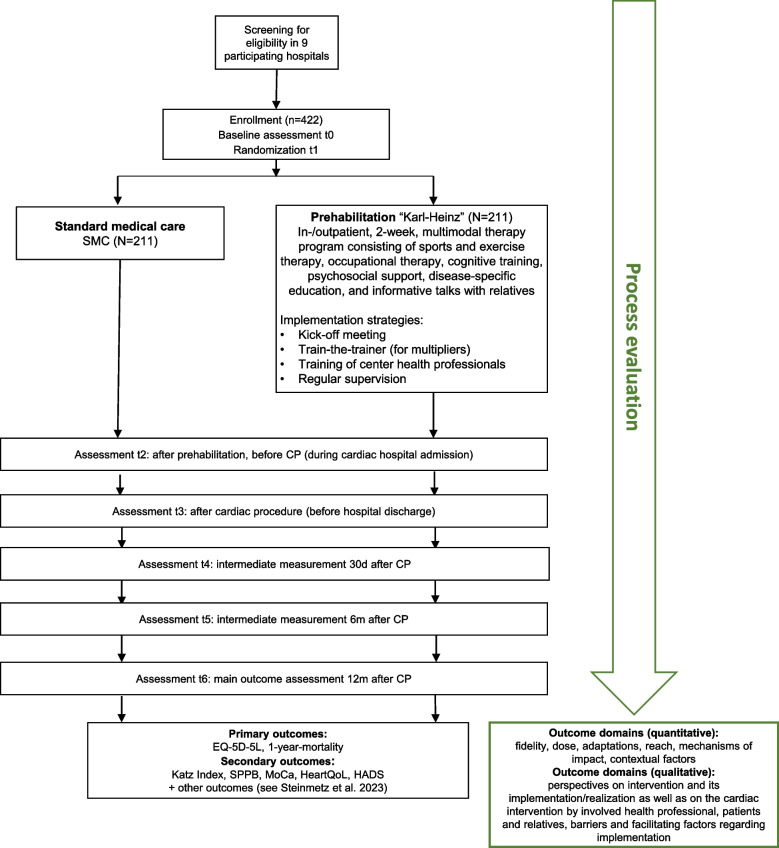
Process evaluation flowchart (green) accompanying the PRECOVERY trial (black). Abbreviations: SMC = standard medical care; CP = cardiac procedure; d = days; m = months; EQ-5D-5L = EuroQol 5-Dimension 5-Level Questionnaire; SPPB = Short Physical Performance Battery; MoCa = Montreal Cognitive Assessment Test; HeartQoL = Heart Quality of Life Questionnaire; HADS = Hospital Anxiety and Depression Scale

PRECOVERY, a randomized, controlled, two-arm parallel group, assessor-blinded, multicenter intervention trial with a total duration of 48 months (http://www.drks.de DRKS00030526, see study protocol [15]), aims to close this gap by employing a multidisciplinary and personalized approach. It evaluates the prehabilitation program “Karl-Heinz” (*K*ognitiv & k*a*rdiale P*r*ehabi*l*itation vor *He*r*zin*terventionen; cognitive and cardiac prehabilitation prior to cardiac procedures), a 2-week, multimodal, inpatient or outpatient prehabilitation program delivered in eight cardiac-specific rehabilitation hospitals (prehabilitation centers) [15]. The program combines physical, cognitive, and educational components to improve QoL and reduce mortality (co-primary outcomes). During the study, all patients undergo a baseline assessment and five follow-up assessments (Fig. 1).

**Fig. 2:**
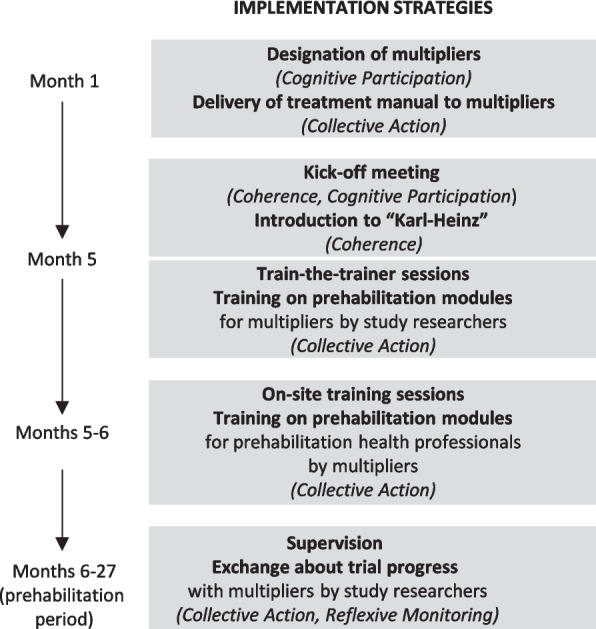
Overview of “Karl-Heinz” implementation strategies, Normalization Process Theory constructs in parentheses [16]

Recruiting centers (cardiology or cardiac surgery departments) and prehabilitation centers are part of the PRECOVERY consortium and thus directly invested in the success of the trial. The “Karl-Heinz” implementation process will begin with a kick-off event for all project partners. One month later, a two-day train-the-trainer event will prepare the change agents (“multipliers”) to facilitate the implementation of the “Karl-Heinz” intervention in each prehabilitation center. Later, multipliers will train the intervention staff in their respective centers in onsite sessions. Regular supervisions between multipliers and researchers of the PRECOVERY study team are planned in the form of online meetings over the entire duration of the prehabilitation period (i.e., the period during which “Karl-Heinz” is performed in the participating prehabilitation centers). See Fig. 2 for an overview of “Karl-Heinz”-implementation strategies and Additional file No. 1 for a more detailed description of “Karl-Heinz” and the implementation strategies.

#### Mixed-methods process evaluation

The PRECOVERY trial is accompanied by a mixed-methods process evaluation in accordance with the Medical Research Council (MRC) Framework for process evaluations of complex interventions [17, 18]. Process evaluations are crucial in complex intervention research, as RCTs alone only demonstrate whether an intervention is effective within a specific time frame and setting. A parallel process evaluation provides insights into the implementation process itself, the underlying implementation mechanisms, and the contextual factors that influence outcomes. This knowledge is essential for interpreting RCT findings and for informing the implementation and adaptation of interventions in other contexts [17, 18].

Consequently, the process evaluation in PRECOVERY aims to provide detailed insights into the implementation and impact of the “Karl-Heinz” intervention. It seeks to generate a comprehensive understanding of the multifaceted factors that influence the implementation and performance of cardiac prehabilitation (Fig. 1). Despite MRC recommendations [17, 18] and the growing number of cardiac prehabilitation trials, process evaluations in this field remain rare; we found only two studies involving a process evaluation in a cardiac prehabilitation trial [19, 20]. Comparable research exists regarding prehabilitation before oncological surgeries, but it is limited in scope and largely qualitative [21–24]. The process evaluation in PRECOVERY addresses these gaps by integrating comprehensive quantitative and qualitative data to generate robust insights into how “Karl-Heinz” is delivered, adapted, and experienced in diverse clinical settings. Ultimately, the findings can provide guidance on how the intervention can be translated and adapted from research settings into routine practice.

#### Program theory and logic model

**Fig. 3:**
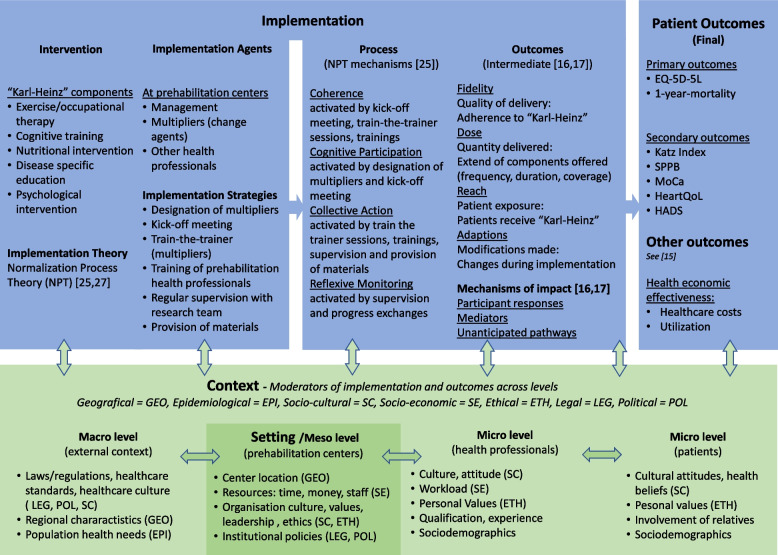
Logic model of “Karl-Heinz.” Abbreviations: EQ-5D-5L (QoL) = Euro Quality of Life; SPPB = Short Physical Performance Battery; MoCa = Montreal Cognitive Assessment Test; HADS = Hospital Anxiety and Depression Scale

A logic model serves as the theoretical foundation for our mixed-methods process evaluation, providing a structured visual representation of our program theory: the hypothesized causal pathways through which the intervention produces outcomes. Our logic model (Fig. 3) was developed iteratively during the proposal phase and before data collection, and has prospectively guided the design of our process evaluation, including the selection of implementation strategies, implementation process assumptions, as well as the identification of contextual factors. The logic model integrates three complementary theoretical frameworks, each addressing different aspects of complex intervention design and evaluation:

1. Context and Implementation of Complex Interventions (CICI) framework [25]: The CICI framework provides the overall design, including implementation, setting, and context. Implementation agents bring an intervention into practice, using specific strategies based on an implementation theory (Normalization Process Theory [16, 26]. The intervention and its implementation are embedded in their context; they may be influenced by contextual moderators across multiple levels. The intervention is implemented in a specific location (setting). Interactions can be found between the setting, implementation, and the overall context.
2. Normalization Process Theory (NPT) [16, 27]: NPT was selected as our implementation theory because it specifically addresses how new practices become routinely embedded in healthcare settings. NPT “proposes that evaluating the implementation of complex interventions requires attention to more than the measurement of outcomes and effectiveness, but also to the social relations and processes related to the *work* that leads to the outcomes. In particular, it guides attention to the processes by which complex interventions are made *workable* and *integrated* into everyday practice” [26]. It provides understanding and explanation of social processes involved in the implementation, embedding, and normalization of an intervention by using four constructs: Coherence, Cognitive Participation, Collective Action, and Reflexive Monitoring [16]. Implementation mechanisms (NPT constructs) explain how “Karl-Heinz” becomes implemented, embedded, and normalized in practice. The implementation strategies of PRECOVERY target all four NPT constructs: *Coherence* is activated through kick-off meetings and intervention introduction*; Cognitive Participation* through designation and engagement of multipliers; *Collective Action* through training sessions, manual delivery, and supervision; and *Reflexive Monitoring* through supervision and progress exchanges. This process evaluation does not establish therapeutic mechanisms of change [28] to explain how the intervention components produce patient outcomes. Our focus remains on implementation processes and mechanisms of impact.
3. MRC Process Evaluation Framework [17, 18]: The MRC framework guides the assessment of implementation outcomes and mechanisms of impact*.* Implementation outcomes (fidelity, dose, reach, adaptations) are assessed following Moore et al. [17, 18] to capture intervention delivery quality and implementation adjustments. Mechanisms of impact reflect how participants respond to and experience the interventions, including the appraisal of their value.

The implementation processes mediate the relationship between successful implementation and patient outcomes. Context, conceptualized through the CICI framework, acts as a moderator throughout, potentially facilitating or inhibiting both implementation and impact. The logic model operationalizes the process evaluation by mapping theoretical constructs to specific, measurable indicators. For fidelity assessment, the logic model specifies: (1) expected implementation processes for each NPT construct; (2) measurable indicators (adherence, modifications); (3) data collection tools (questionnaires, therapy plans, patient diaries); and (4) analytical triangulation of quantitative measures with qualitative data on barriers. When deviations are detected, the logic model's theoretical structure guides the investigation into root causes—whether issues stem from insufficient activation of specific NPT constructs [16] or from contextual factors at macro, meso, or micro levels [25]. This systematic, theory-driven approach allows us to identify not only where fidelity wavered but also why, informing targeted improvement strategies. These insights are essential for understanding implementation success, refining intervention strategies, optimizing resource allocation, and enhancing reproducibility and scalability in real-world settings [17, 29, 30].

### Objectives 

Following the recommendations from existing frameworks for process evaluations of complex interventions [17, 29, 30], the main objective of the process evaluation is to examine the implementation of the “Karl-Heinz” intervention in the PRECOVERY trial while also considering the reactions and perspectives of the stakeholders involved. The following research questions will be assessed:

1. How is the intervention “Karl-Heinz” implemented across prehabilitation centers? What contextual factors facilitate or impede the implementation?
2. What is the intervention’s quality in terms of fidelity, dose, adaptations, and reach of “Karl-Heinz”?
3. What are the key stakeholders’ perspectives on the implementation and perceived impact of “Karl-Heinz”?

Research question (1) examines how “Karl-Heinz” is embedded and normalized across sites (Normalization Process Theory [16, 27]). Specifically, how and to what extent do health professionals (all professionals who are in direct contact with the PRECOVERY patients, such as nurses, therapists, and physicians) respond to, accept, and integrate the intervention into their work-related routines? Research question (2) analyzes the effects of implementation quality and contextual factors on the primary outcomes of the PRECOVERY trial, examining five key dimensions: “Fidelity” assesses the quality of intervention delivery—the degree to which health professionals deliver “Karl-Heinz” as intended, including adherence to core components and delivery competence. Since “Karl-Heinz” is an entirely new prehabilitation program, the process evaluation is exploratory in design, and thresholds for fidelity cannot be determined beforehand. “Dose” examines whether the modules of the intervention are offered to the planned extent (quantity of intervention delivered). By investigating “adaptations,” we track how “Karl-Heinz” is delivered in practice and the modifications that occur. We distinguish between changes that maintain core intervention principles and those that potentially undermine fidelity. “Reach” captures patient-level exposure and describes how and to what extent the patients engage with the intervention (utilization of treatments). Finally, we understand “context” as organizational, provider, and patient-level factors external to the intervention that may facilitate or impede implementation mechanisms and quality (fidelity, dose, adaptations, reach) [17, 18]. Research question (3) examines the opinions of patients, relatives, and health professionals on the intervention modules and the intervention as a whole.

The quantitative part of the process evaluation will address all three research questions above. The qualitative part focuses on analyzing the perspectives of the involved stakeholders (patients, their relatives, prehabilitation center health professionals, physicians performing the cardiac procedure, and general practitioners). By triangulating results from both the quantitative and qualitative strands of the process evaluation, we will develop a comprehensive understanding that addresses all research questions. Moreover, we will derive recommendations on how to introduce “Karl-Heinz” into standard care, including any necessary adaptations.

### Design 

The mixed-methods process evaluation includes a quantitative and a qualitative strand and will be conducted in parallel to the main trial (Fig. 1). The Department of General Practice, University Medical Center Göttingen (UMG) is responsible for organizing and carrying out the process evaluation. Quantitative data will be analyzed by the Department of Medical Statistics, UMG; qualitative data will be evaluated by the Department of General Practice, UMG*.* The findings will be combined and triangulated to provide a comprehensive understanding of the results of the main trial.

## Methods: participants, interventions, and outcomes

### Study setting 

The PRECOVERY trial is conducted in multiple recruiting centers across Lower Saxony, Baden-Württemberg, and Brandenburg, Germany, all providing maximum-care cardiac services and ensuring timely patient recruitment based on their historical procedure volumes. Lower Saxony centers were required to treat a representative number of patients insured by AOK Lower Saxony, a major statutory health insurance provider and a key partner in PRECOVERY (see Additional file No. 2). AOK Lower Saxony will supply routine insurance data on healthcare costs for enrolled patients.

Eight prehabilitation centers were selected according to the following criteria: cardiac specialization, prior collaboration with the recruiting centers, geographic proximity to recruiting centers, and infrastructure capacity to deliver all components of “Karl-Heinz.” Seven prehabilitation centers provide inpatient prehabilitation, while one (Ulm) provides outpatient prehabilitation.

Data collection for the process evaluation primarily takes place in inpatient settings (see Additional file No. 2) with additional data collection from patients and relatives in both groups before (patients only) and after the cardiac procedure, as well as during follow-up in outpatient settings.

**Fig. 4:**
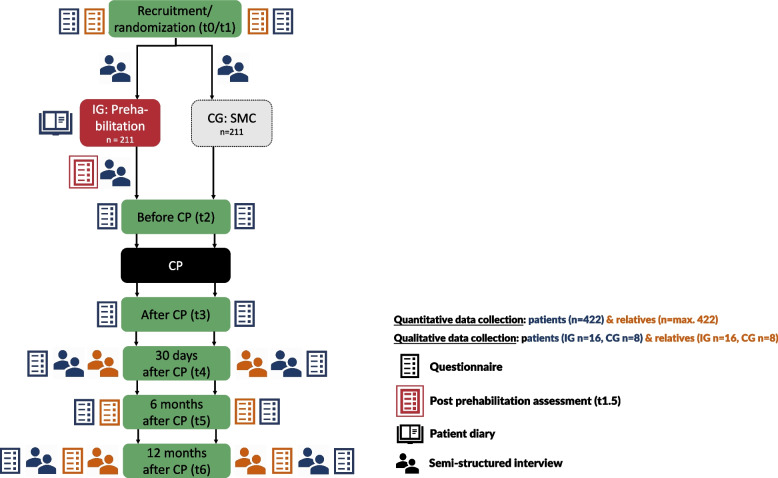
Schedule of patient and relative enrollment, intervention, and assessments for the process evaluation. Blue: patients’ assessments, orange: relatives’ assessments. Abbreviations: IG = intervention group, CG = control group, SMC = standard medical care, CP = cardiac procedure

**Fig. 5:**
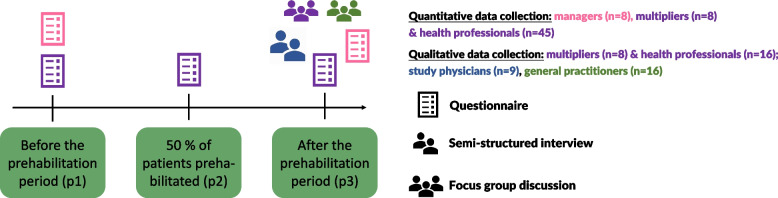
Quantitative and qualitative data collection points for prehabilitation center managers, multipliers, and other health professionals, study physicians, and general practitioners. Pink: managers of prehabilitation centers, purple: multipliers and prehabilitation center health professionals, blue: study physicians, green: general practitioners

Qualitative data collection will take place from a subsample of patients and relatives in both settings (see Fig. 4). Additionally, study physicians from the cardiac recruitment centers (inpatient care) and GPs (outpatient care) will also be involved (see Fig. 5). Reporting will follow the Good Reporting of A Mixed Methods Study (GRAMMS) criteria [31]. The “Karl-Heinz” intervention is described in Additional file No. 1 according to the Template for Intervention Description and Replication (TIDieR) criteria [32]. Table 2 provides the schedule of enrollment, intervention, and assessments.

### Eligibility criteria 

Patients included in the process evaluation meet the same eligibility criteria as for the PRECOVERY trial: age ≥ 65 years and scheduled for elective cardiac procedures defined by the German operation and procedure codes catalog (OPS) (see Tables 1 and 2 in the PRECOVERY study protocol [15]). There are no specific inclusion or exclusion criteria for participating relatives. All PRECOVERY patients automatically take part in the quantitative strand. For the qualitative strand, interested patients and relatives will be selected using maximum variation sampling regarding sex, age, enrollment location, residence (rural/urban), and level of care. Multipliers are designated by the prehabilitation center management. Other prehabilitation center health professionals are recruited by multipliers based on (1) providing patient care during prehabilitation, (2) having completed multiplier-led training, and (3) presumed employment during the intervention period. GPs must care for at least one intervention group patient during the 12-month follow-up period. Study physicians are designated by their respective management. Table 1 provides an extensive overview of data sources and measurement points by stakeholder groups.

**Table 1:** Overview of data sources, measuring points, and sample sizes by target groups for the process evaluation (quantitative and qualitative)

Target population	Data collection	Measurement points												Target population and recruitment strategies	Sample size
		**Train-ing**	**t**_**0**_**/t**_**1**_	**During preha**	**t**_**1.5**_	**t**_**2**_	**t**_**3**_	**t**_**4**_	**t**_**5**_	**t**_**6**_	**p**_**1**_	**p**_**2**_	**p**_**3**_		
Patients (intervention group)	Quantitative		X (Q)	X (PD)	X (PPA)	X (Q)	X (Q)	X (Q)	X (Q)	X (Q)				Patients fulfilling the inclusion and exclusion criteria will be asked for participation in the PRECOVERY trial (for a complete list of the criteria, see [15]). Information on PE-data collection is included in the informed consent for the PRECOVERY trial. Consenting to take part in the PRECOVERY trial includes consenting to the quantitative PE	*n* = 211
	Qualitative		X (I)		X (I)			X (I)		X (I)				Participation in the qualitative PE is voluntary, and an additional informed consent form is used for confirmation. A subsample is selected by the PE-team applying a maximum variation approach regarding sex, age, place of enrollment, place of residence (rural or urban), and level of care	*n* = 16
Patients (control group)	Quantitative		X (Q)			X (Q)	X (Q)	X (Q)	X (Q)	X (Q)				Patients fulfilling the inclusion and exclusion criteria will be asked for participation in the PRECOVERY trial (for a complete list of the criteria, see [15]. Information on PE-data collection is included in the informed consent for the PRECOVERY trial. Consenting to take part in the PRECOVERY trial includes consenting to the quantitative PE	*n* = 211
	Qualitative		X (I)					X (I)		X (I)				Participation in the qualitative PE is voluntary, and an additional informed consent form is used for confirmation. A subsample is selected by the PE-team applying a maximum variation approach regarding sex, age, place of enrollment, place of residence (rural or urban), and level of care	*n* = 8
Relatives (intervention group)	Quantitative		X (Q)						X (Q)	X (Q)				Relatives of study patients will be asked for participation in the PRECOVERY trial [15]. Information on PE-data collection is included in the informed consent for the PRECOVERY trial. Consenting to take part in the PRECOVERY trial includes consenting to the quantitative PE	*n* = max. 211
	Qualitative							X (I)		X (I)				Participation in the qualitative PE is voluntary, and an additional informed consent form is used for confirmation. A subsample is selected by the PE-team applying a maximum variation approach regarding sex, age, place of enrollment, place of residence (rural or urban), and level of care	*n* = 16
Relatives (control group)	Quantitative		X (Q)						X (Q)	X (Q)				Relatives of study patients will be asked for participation in the PRECOVERY trial [15]. Information on PE-data collection is included in the informed consent for the PRECOVERY trial. Consenting to take part in the PRECOVERY trial includes consenting to the quantitative PE	*n* = max. 211
	Qualitative							X (I)		X (I)				Participation in the qualitative PE is voluntary, and an additional informed consent form is used for confirmation. A subsample is selected by the PE-team applying a maximum variation approach regarding sex, age, place of enrollment, place of residence (rural or urban), and level of care	*n* = 8
Managers of prehabilitation centers	Quantitative										X (Q)		X (Q)	Participation for the managers of the prehabilitation centers who are also members of the PRECOVERY consortium is mandatory	*n* = 8
Multipliers	Quantitative	X (E, P)		X (PD)							X (Q)	X (Q)	X (Q)	Multipliers are designated by the respective managers of the prehabilitation center and have neither inclusion nor exclusion criteria. Participation for all multipliers is mandatory	*n* = 8
	Qualitative												X (FG)	Participation in the qualitative PE is voluntary. Multipliers are asked to participate by the PE team. Multipliers have neither inclusion nor exclusion criteria and are contacted by the PE-team via telephone	*n* = 8 (1 FG)
Involved prehabilitation center health professionals l (e.g., therapists, nurses,physicians)	Quantitative	X (E)		X (PD)							X (Q)	X (Q)	X (Q)	Prehabilitation center health professionals are recruited by the local multiplier according to the following recommended inclusion criteria: 1. caring for prehabilitation patients, 2. previously trained by a multiplier, and 3. presumable employment during the entire duration of the intervention period	Q: *n* = 40 (5 per center)
	Qualitative												X (FG)	Participation in the qualitative PE is voluntary. Prehabilitation center health professionals are recruited by the local multiplier according to the following recommended inclusion criteria: 1. caring for prehabilitation patients, 2. previously trained by a multiplier, and 3. presumable employment during the entire duration of the intervention period	*n* = 16 (2 per center, 8 per FG)
Study researchers	Quantitative	X (P)												Participation for the study researchers who are also members in the PRECOVERY consortium is mandatory	*n* = 3
Study physicians	Qualitative												X (I)	Study physicians performing the cardiac procedures are invited by letter and telephone by the PE-team and have neither inclusion nor exclusion criteria	*n* = 9
General practitioners	Qualitative												X (FG)	Names of GPs are given by patients during their enrollment. Inclusion criteria for GPs is to provide medical care for at least one IG-patient during the 12 months after the cardiac procedure. GPs are invited by letter and telephone by the PE-team and informed consent is given before participation	*n* = 16 (8 per FG)

*Abbreviations*: *t*_*1*_ = randomization/baseline, *t*_*1.5*_ = after prehabilitation, *t*_*2*_ = before cardiac procedure, *t*_*3*_ = after cardiac procedure, *t*_*4 =*_ 30 days after cardiac procedure, *t*_*5*_ = 6 months after cardiac procedure, *t*_*6*_ = 12 months after cardiac procedure, *p*_*1*_ = before the prehabilitation period, *p*_*2*_ = after about 50% of patients are prehabilitated, *p*_*3*_ = after the prehabilitation period, *preha* = prehabilitation “Karl-Heinz”, *PE* = process evaluation, *Q* = questionnaire, *IG* = intervention group, *PD* = patient diary, *PPA* = post-prehabilitation assessment, *E* = evaluation, *P* = protocol, *I* = semistructured telephone interview, *FG* = online focus group discussion

**Table 2:**
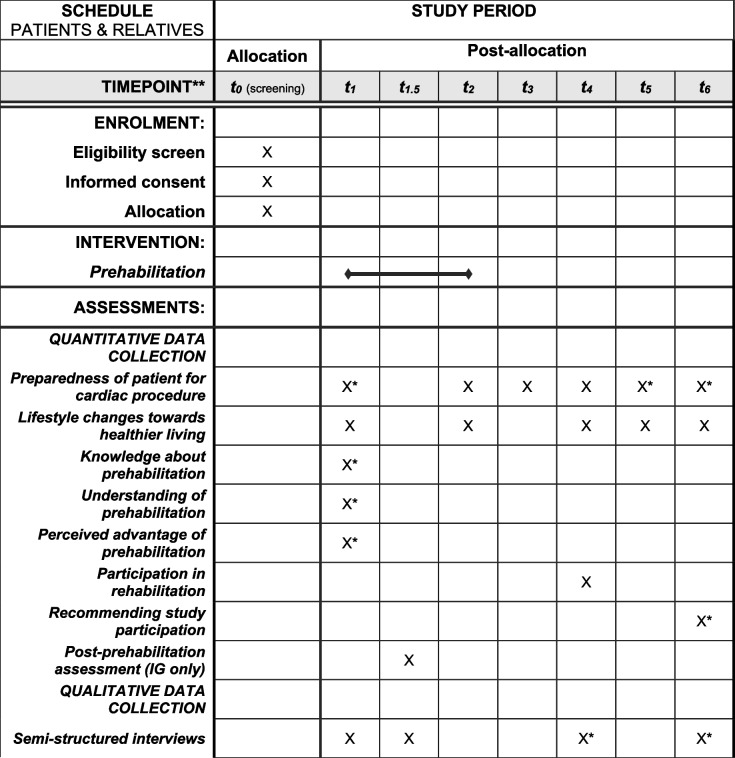
Schedule of enrolment, intervention, and assessments

*t*_*1*_ = randomization/baseline, *t*_*1.5*_= after prehabilitation, *t*_*2*_ = before cardiac procedure, *t*_*3*_ = after cardiac procedure, *t*_*4*_ = 30 days after cardiac procedure, *t*_*5*_ = 6 months after cardiac procedure, *t*_*6*_ = 12 months after cardiac procedure, *IG* intervention group

^*^ Assessments for both patients and their relatives

^**^ For the qualitative process evaluation, multipliers and prehabilitation center health professionals give informed consent to participate in focus groups, study physicians for semi-structured interviews toward the end of the recruitment period. Prehabilitation center managers, multipliers, and health professionals also answer questionnaires before the prehabilitation period (p1), after about 50% of patients are prehabilitated (p2; multipliers and health professionals only), and after the prehabilitation period (p3)

### Who will take informed consent? 

Study physicians at the recruiting centers will obtain informed consent from patients and relatives for the PRECOVERY trial. The process evaluation team will obtain additional informed consent for qualitative participation from all stakeholder groups.

### Participant timeline 

Figure 4 outlines data collection from patients and relatives across the 14-month study period (t0/t1–t6). Figure 5 provides an overview of the data collection from health professionals and study physicians at prehabilitation centers, and GPs across the 22-month prehabilitation period (p1–p3). 

### Sample size 

The PRECOVERY trial includes 422 patients randomized 1:1 (see [15]). Sample sizes for the process evaluation are described in Table 1.

For quantitative analyses of the process evaluation, all intervention patients (*n* = 211) will provide comprehensive data. Control group patients (*n* = 211) and relatives of both groups (*n* = max. 422) will answer selected questions. Moreover, data will be collected from managers of the eight prehabilitation centers, all training participants, and additionally from five health professionals per center.

For the qualitative analyses, 24 interviews with patients and 24 with relatives will be conducted (16 from the intervention group, 8 from the control group). Based on the literature, saturation will be reached after 13–20 interviews [33]. Nine study physicians (one per recruiting center) will be interviewed. Moreover, three focus groups will be conducted with multipliers and other health professionals of the prehabilitation centers, consistent with recommendations of performing at least three focus groups per person group [34]. Two focus groups with GPs are a realistic target given the expected recruitment rates of about 5–10%.

### Recruitment 

Process evaluation participants are enrolled through the main trial [15]. After providing informed consent, the process evaluation team contacts patients and relatives by telephone to confirm participation and to schedule interviews. Prehabilitation centers and the recruiting centers belong to the PRECOVERY consortium, enabling direct contact with managers, multipliers, and study physicians.

Multipliers invite additional health professionals in the prehabilitation centers to participate. GPs are identified through information collected during patient enrollment and are invited by letter by the process evaluation team. A detailed overview of the recruitment strategies is provided in Table 1.

### Data collection and management

#### Quantitative data

The quantitative data collection focuses on four major outcome domains: (1) implementation strategies and activities, (2) the intervention package “Karl-Heinz,” (3) patient assessments (preparedness for cardiac intervention and lifestyle changes), and (4) contextual factors. These domains are measured across several dimensions, including dose, reach, adaptations, expectations, attitudes, quality, and satisfaction. Table 3 provides an overview of outcomes measured via quantitative methods based on our logic model (Fig. 3).

**Table 3:** Overview of the outcome domains and subdomains of the quantitative process evaluation over the course of the study

Domains	Subdomains	Dimensions	Measurement instruments	Target population and measurement points								
**Implementation strategies and activities**				**Patients** (intervention group)	**Patients** (control group)	**Relatives** (intervention and control group)	**Managers**	**Multipliers**	**Preha health professio-nals**	**Study** **researchers**	**Study** **physicians**	**General** **practitioners**
Implementation strategies	Kick-off meeting	Attitudes, quality, satisfaction, contextual factors	Questionnaires	-	-	-	Before first patient in	-	-	Before first patient in	-	-
	train-the-trainer event (training of multipliers)	Attitudes, quality, satisfaction, contextual factors	Protocols, questionnaires	-	-	-	-	Before first patient in	-	Before first patient in	-	-
		Reflection of own role, motivation for participation, preparedness, satisfaction with informational material, barriers, and facilitating factors	Questionnaires	-	-	-	-	Before first patient in	-	-	-	-
	training of preha health professionals involved in “Karl-Heinz”	Attitudes, quality of training, and satisfaction	Protocols, questionnaires	-	-	-	-	Before first patient in	Before first patient in	-	-	-
		Reflection of own role, motivation for participation, preparedness, satisfaction with informational material, work experience, barriers and facilitating factors	Questionnaires	-	-	-	-	-	Before first patient in	-	-	-
Implementational work within preha centers	Coherence, cognitive participation, collective action, reflexive monitoring	Fidelity, context, adaptations, attitudes, quality, and satisfaction	Questionnaires (Normalization measure development questionnaire)	-	-	-	p1, p3	p1, p2, p3	p1, p2, p3	-	-	-
Supervision during preha period	Center specific barriers and facilitating factors, organization, available resources, forms of collaboration		Minutes	-	-	-	-	Monthly	-	-	-	-
**Intervention package “Karl-Heinz”**												
Intervention package as a whole		Fidelity, context, adaptations, expectations, attitudes, scope and design, quality, satisfaction	Questionnaires (Normalization measure development questionnaire); post-prehabilitation assessment	t1.5	-	-	p1, p3	p1, p2, p3	p1, p2, p3	-	-	-
Sessions as part of the intervention package “Karl-Heinz”	Sports and exercise therapy, occupational therapy, cognitive training, psychosocial support, disease-specific training, informative talks with relatives, hygiene training/nutritional intervention	Fidelity, dose, adaptations, reach	Patient diary, patients’ therapy plans	During preha	-	-	-	During preha	During preha	-	-	-
**Assessments over the course of participation in the study**												
Assessment of preparedness for cardiac intervention and changes in lifestyle			Questionnaires	t0/t1, t2, t3, t4, t5, t6	t0/t1, t2, t3, t4, t5, t6	t0/t1, t5, t6	-	-	-	-	-	-
**Contextual factors**												
Setting/meso level (preha centers + regarding intervention)	Center location, organization, work environment specific barriers and facilitating factors,	Context	Questionnaires	-	-	-	p1, p3	p1, p2, p3	p1, p2, p3	-	-	-
	Institutional Policies, Resources (time, money, staff)	Context	Questionnaires	-	-	-	p1, p3	-	-	-	-	-
Micro level (regarding health professionals l)	Culture, attitude, personal values, level of qualification, adaptations	Context	Questionnaires	-	-	-	p1, p3	p1, p2, p3	p1, p2, p3	-	-	-
Micro level (regarding patients of intervention group at preha center)	Health beliefs, involvement of relatives, sociodemo-graphics	Context	Post-prehabilitation assessment, questionnaires	t1.5	-	t5	-	-	-	-	-	-
	Admission to/discharge from preha center, nursing/medical care, rounds, accommodation and meals, contact with other patients	Context	Post-prehabilitation assessment	t1.5	-	-	-	-	-	-	-	-

*Abbreviations*: *preha* prehabilitation “Karl-Heinz”, *p1* = before prehabilitation period, *p2* = during prehabilitation period, *p3* = after prehabilitation period

^*^ Single items or subscales of reported tools are used for the outcome domains in question. In addition, self-developed items are used for the measurement of all outcome domains

Data of trial patients and their relatives is collected along their individual timeline of participation (Fig. 4; see Additional file No. 3 for a detailed overview of the questionnaires used and Additional file No. 4 for a translated version of the patient diary). Study nurses in the recruiting centers collect questionnaires from patients and their relatives (from t0/t1 until t6, *n* = max. 844) face-to-face or by telephone. The process evaluation team collects patient diaries, therapy plans, and conducts post-prehabilitation telephone assessments with intervention patients (*n* = 211) using an adapted version of the Hamburg Questionnaire on Hospital Stay [35] combined with adapted questions from the German Pension Insurance quality assurance program [36].

During the 22-month prehabilitation period, the process evaluation team monitors the implementation, collects protocols from the train-the-trainer events, and gathers evaluation forms from multipliers and health professionals after their training. Prehabilitation center managers complete questionnaires before and after the prehabilitation period (p1, p3), which describe the specifics of their center (see Fig. 5). In addition, multipliers and health professionals complete questionnaires at up to three timepoints (p1, p2, p3). The Normalization Measure Development (NoMAD) questionnaire [37] is part of the latter questionnaires, which evaluate implementation processes using NPT constructs.

#### Qualitative data

Qualitative data will be collected via semi-structured telephone interviews with patients and relatives at up to four timepoints (see Fig. 4) and with study physicians at one timepoint (see Fig. 5). Online focus groups will be conducted with multipliers (*n* = 1), other health professionals (*n* = 2), and GPs (*n* = 2). Interview guides will be theoretically informed and incorporate findings from previous interviews. Table 4 provides an overview of topics covered. Guides will be piloted in advance to ensure an appropriate timeframe as well as question clarity. All data will be collected by trained members of the process evaluation team. The recordings of interviews and focus groups will be transcribed verbatim and then pseudonymized.

**Table 4:** Overview of topics covered in interviews and focus groups of the qualitative process evaluation

Data collection method	Themes
Semistructured interview	
With patients	For patients in the intervention and control group: • View of the upcoming cardiac procedure • Perspective on the post-procedure period • Wishes regarding health outcomes • Lifestyle changes (nutrition, exercise, etc.) Additionally, only for intervention patients: • Acceptance, implementation, and evaluation of prehabilitation • View of prehabilitation implementation, reactions, and adjustments • Impact of prehabilitation on the experience of the procedure • Satisfaction with prehabilitation and perceived benefits • Identification of barriers and facilitators in the implementation
With relatives	For relatives in the intervention and control group: • Perspective on the upcoming cardiac procedure • View of the post-procedure period • Health outcome wishes Additionally, only for intervention relatives: • Acceptance, implementation, and evaluation of prehabilitation • Impact of prehabilitation on their own well-being and the patient • Perception of the family discussion during prehabilitation • Satisfaction with prehabilitation • Identification of barriers and facilitators in prehabilitation
With study physicians	• View of their own role • Perspective on outpatient consultations • View of procedures • Perception of patients' health progress • Assessment of the usefulness of prehabilitation • Suggestions for improvements, wishes, and advice to the study team
Focus group discussion	
With multipliers and prehabilitation center health professionals	• View of the implementation of prehabilitation in the facility • Perspective on their own role • View of patients receiving prehabilitation • Assessment of the usefulness of prehabilitation • Suggestions for improvements, wishes, advice to the study team
With GPs of IG-patients	• View of the implementation of prehabilitation • Perspective on patients receiving prehabilitation • Assessment and evaluation of the usefulness of prehabilitation • Suggestions for improvements, wishes, advice to the study team

*Abbreviations*: *GP* = general practitioner, *IG* = intervention group

#### Data management 

Upon enrollment, each patient is assigned a pseudonymization code. For all data collection, only the pseudonymization code will be used (see [15] for additional information). Training protocols from the train-the-trainer event and the on-site training will be collected in pseudonymized form. Training evaluations from other health professionals will be collected anonymously. Incoming patient diaries and therapy plans will be checked for completeness by the process evaluation team and then pseudonymized before being entered into REDCap. Multipliers will be contacted if entries are missing or ambiguous. Post-prehabilitation assessment data will either be collected on paper or entered directly into the database. A second team member will review all entered data.

#### Confidentiality 

Quantitative data will be recorded on paper, in REDCap, or on electronic devices, treated confidentially, and transmitted only in pseudonymized or anonymized, encrypted, and password-protected form. Access to the original documents will be denied to third parties. Participants in the qualitative process evaluation will receive additional individual pseudonymized IDs, which will be kept separately from the collected data. Retrospective identification is possible only with a key, accessible only to the process evaluation team. Audio recordings, contact details, and pseudonymization lists will be deleted after the study ends. All other data will be stored for 10 years. A data privacy statement was approved by the UMG data protection official.

### Data analyses

#### Quantitative data

The analysis of the quantitative data from the process evaluation will begin after the last patient completes prehabilitation. For all measures described, descriptive statistics will be reported. The confirmatory statistical analysis of the main trial will be conducted, focusing on the primary outcomes (QoL and 1-year mortality) [15] using a joint frailty model for longitudinal data and terminal event [38].

Exploratory sensitivity analyses will explore how fidelity, dose, reach, adaptations, and contextual factors might influence the study outcomes. We will assess the potential interactions of these factors with the treatment effect by including interaction terms and the coefficient for the treatment effect (treatment-time interaction term) in the frailty model. In addition to the intention-to-treat analysis, which is the primary analysis strategy for both the PRECOVERY trial and the process evaluation, we will also examine a population of participants who have started and adhered to the intervention as planned (starting and adhering estimand). Missing data will be replaced by multiple imputation. Analyses will be performed using R.

#### Qualitative data

Qualitative data will be analyzed by two researchers using qualitative content analysis by Kuckartz [39] with MAXQDA 2024, working either collaboratively or consecutively. Themes and patterns related to implementation and stakeholder perspectives will be identified through deductive and inductive coding, developed from analyzing a subsample of interviews and focus groups. Findings will be discussed in regular team meetings.

#### Integration and triangulation

The mixed-methods design allows integration of findings into the development of data collection instruments. In particular, interview guides for the focus groups with multipliers and health professionals will be developed during the study, taking into account observations from the process evaluation to validate theoretical assumptions and issues that arose during the study period.

Data analysis will be conducted in three steps. First, quantitative and qualitative data will be analyzed separately, with results from each strand explaining findings and providing supplementary contextual information for the other strand. Second, joint display analysis will triangulate the data, validating and corroborating the findings, and optimizing the understanding of combined results [40]. Third, after the main trial analysis is finalized, the process evaluation results will be employed to review trial outcomes and to demonstrate connections between implementation and patient outcomes. These analyses will identify areas of wavering intervention fidelity, explain differing levels of participant engagement, and determine the role of contextual factors in shaping intervention delivery.

#### Dissemination plans 

Results will be disseminated through peer-reviewed publications in medical journals, national and international conferences, and a study report to the Federal Joint Committee (G-BA). A summary report will be provided to all partners and made available on the project homepage. Authorship will adhere to the recommendations of the International Committee of Medical Journal Editors [41].

## Discussion

The mixed-methods process evaluation accompanying the PRECOVERY trial, which is guided by the MRC framework for process evaluations of complex interventions [17, 18, 30], tracks multiple variables moderating and mediating the delivery of the “Karl-Heinz” intervention and its impact on key outcomes. It collects extensive quantitative and qualitative data from multiple stakeholders prior to prehabilitation, during prehabilitation, after the cardiac procedure, and during the 12-month follow-up.

Our comprehensive design differs from existing process evaluations in three key ways. First, unlike studies focusing on adherence and implementation [22], we delve deeper into the contextual factors. Second, while other studies collect data only at one timepoint [19, 42] we will recruit process evaluation participants throughout the entire prehabilitation period, beginning with the initial training and ending after the last patient has left the prehabilitation center. Furthermore, we collect data immediately after patients receive the “Karl-Heinz” intervention to capture immediate rather than retrospective experiences. Third, we include a broader range of stakeholders (similar to the design in [21, 43]) to provide a holistic understanding of the intervention’s impact in medical practice.

Furthermore, the mixed-methods approach offsets the limitations of single data collection methods. Combining quantitative data from all patients and relatives with qualitative data from a subsample enriches and explains findings. Triangulation across multiple sources (patients, relatives, healthcare professionals, and GPs) will further enhance credibility and offer a nuanced understanding of intervention delivery and perception.

However, detailed documentation may burden health professionals and create a sense of being monitored. Although a pilot phase was not planned for the PRECOVERY trial, we expect the individual time burden to be limited to 10 min per questionnaire, with more extensive data collection restricted to multipliers as part of their designated tasks. Conversely, detailed documentation might improve adherence, as health professionals are aware of the need to document and justify deviations from the planned intervention.

Selection bias is another potential limitation, as participating patients and individual staff members of the prehabilitation centers may have a greater interest in the prehabilitation and its success. To address this, we will collect data from a diverse sample across different backgrounds and experiences within the staff of the prehabilitation centers. However, selection bias cannot be completely avoided. To minimize interviewer bias, we will train researchers in a standardized way including a probing interview to ensure appropriate data collection techniques. Additionally, the team will reflect regularly on the processes of the interviews and perspectives of interviewers. Two researchers will independently code qualitative data with regular discussions of results among the team.

The findings of the process evaluation will strengthen the results of the PRECOVERY trial by offering insights explaining its results and possible optimizations of the “Karl-Heinz” intervention. The findings will enable refinement of future cardiac prehabilitation programs in Germany and, to some extent, internationally, to better align with patients’ and providers’ needs. While generalizability beyond cardiac prehabilitation is limited, the findings may still inform prehabilitation programs for other conditions. Ultimately, our findings should support the integration of this new form of care into routine practice in the German healthcare system, thus contributing to improved care for older cardiac patients.

## Trial status

After the kick-off meeting on February 16th, 2023, the final staff training was conducted on March 16th and 17th, 2023, and the first patient was randomized in April 2023. Recruitment was completed in May 2025. The follow-up phase of PRECOVERY will be finalized by the end of July 2026. Recruitment and data collection for the mixed-methods process evaluation are ongoing. The first interview with a patient recruited for the qualitative part of the process evaluation was conducted in June 2023. Recruitment of patients and their relatives was completed in May 2025; the last professional participants (GPs) will be recruited in summer 2026. Data collection for both the quantitative and qualitative strands of the process evaluation is expected to be finalized in the third quarter of 2026.

## Supplementary Information

Additional file 1. Description of the intervention “Karl-Heinz” following the Template for Intervention Description and Replication (TiDier) criteria [32].

Additional file 2. Complete list of recruiting centers, prehabilitation centers, and scientific partners of PRECOVERY.

Additional file 3. Overview of standardized instruments used for the quantitative part of the process evaluation.

Additional file 4. “Karl-Heinz”-Diary for patients and health professionals.

## Data Availability

The data is available upon reasonable request to the publication committee of the PRECOVERY project. The results of the process evaluation will be reported in scientific journals and conference presentations. The findings will be used to provide context for the results of PRECOVERY and to improve the implementation of the complex intervention for standardized care for this group of patients in Germany. We acknowledge support by the Open Access Publication Funds of the Göttingen University.

## Abbreviations

CG: Control group
CP: Cardiac procedure
DRKS: German Clinical Trial Register
E: Evaluation
EQ-5D-5L (QoL): EuroQol 5-Dimension 5-Level Questionnaire
FG: Online focus group discussion
G-BA: Federal Joint Committee
GP: General practitioner
HADS: Hospital anxiety and depression scale
HeartQoL: Heart Quality of Life Questionnaire
I: Semistructured telephone interview
IG: Intervention group
MoCa: Montreal Cognitive Assessment Test
MRC: Medical Research Council
NoMAD: Normalization measure development questionnaire
P: Protocol
PD: Patient diary
PE: Process evaluation
PPA: Post-prehabilitation assessment
Preha: Prehabilitation “Karl-Heinz”
p1: Before prehabilitation period
p2: During prehabilitation period
p3: After prehabilitation period
Q: Questionnaire
QoL: Quality of life
SPPB: Short Physical Performance Battery
SMC: Standard medical care
TtT: Train the trainer
UMG: University Medical Center Göttingen
