# Supplementary material for: Mixed-methods process evaluation of the “Karl-Heinz” cardiac prehabilitation program in older patients: protocol for the PRECOVERY trial
Source: Trials. 2026 Mar 18;27:283. doi: 10.1186/s13063-026-09608-4 (PMC13063718; doi:10.1186/s13063-026-09608-4)
Supplement: Supplementary file 1 — Additional file 1. Description of the intervention “Karl-Heinz” following the Template for Intervention Description and Replication (TiDier) criteria [32]. [file 13063_2026_9608_MOESM1_ESM.docx]

## Additional file No. 1

**Description of the intervention “Karl-Heinz” following the Template for Intervention Description and Replication (TiDier) criteria [35]**

**1. Brief Name**

PRECOVERY trial: Prehabilitation in older patients prior to elective cardiac procedures.

**2. Why**

The implementation of the prehabilitation program “Karl-Heinz” aims to improve the QoL, long-term survival, and functional capacity of patients aged 65 years or older undergoing a cardiac procedure [15]

**3./4. What (Materials/Procedures)**

The two-week multimodal “Karl-Heinz” intervention fulfills the criteria of a complex intervention since it contains seven fixed modules (see Table 2), which are tailored individually to each patient after an initial assessment by the responsible study physician of the prehabilitation center. The seven modules and a set of minimum required therapies are described in the open source publication ([15]: table 3, additional file No. 3).

The implementation of the “Karl-Heinz”-intervention was facilitated by a few strategies:

Table 1: Strategies designed for the implementation of the prehabilitation program “Karl-Heinz”

| **Time point** | **Description of the activity** | **Target persons** | **Persons responsible for delivery or application** | **Location** | **Modes of delivery** | **Frequency, dosage, and duration** | **Materials used**  **(provided by the study researchers)** |
| --- | --- | --- | --- | --- | --- | --- | --- |
| Before and during the writing of the proposal | Building of the PRECOVERY study group | Management of prehabilitation centers | Project leader of PRECOVERY | Not applicable | E-Mail, video-calls, telephone,  face-to-face contacts | Not applicable | Written overview of study goals and design, PowerPoint Presentations |
| During the writing of the proposal | Development of the treatment manual of “Karl-Heinz” | Management of prehabilitation centers, study researchers, and experts | Project leader of PRECOVERY and research team | Not applicable | E-Mail, workshop (video) | 2 days, 3h/day | Draft of treatment manual, PowerPoint Presentation |
| At the beginning of the study | Kick-off meeting | Management of prehabilitation centers, multipliers, management, and study nurses of recruiting centers | Project leader of PRECOVERY and study researchers | University Medical Center Göttingen | Conference (face-to-face) | 1 day,  5 hours | Talks, PowerPoint Presentations, Discussion |
| At the beginning of the study | Training of multipliers | Multipliers | Project leader of PRECOVERY and study researchers | University Medical Center Göttingen | Workshop (face-to-face) | 1 day,  8 hours | Treatment manual, checklists, videos, recorded talks of the kick-off meeting, PowerPoint Presentations |
| Before first patient in prehabilitation centers | Training of health professionals involved in “Karl-Heinz” | Health professionals in prehabilitation centers | Multipliers | Prehabilitation centers | Training | Duration as needed; typically,  ~1 hour per training | Treatment manual, checklists, videos, and recorded talks of the kick-off meeting |
| During prehabilitation period | Supervision | Multipliers | study researchers | Not applicable | Video-conference | Monthly,  1 hour, if necessary more often | PowerPoint Presentation, discussion |

**Kick-off meeting for management and coordinating team members of prehabilitation facilities**

Before the kick-off meeting, all managers of the prehabilitation centers designate one “multiplier” (change agent) for their center. The multipliers are responsible for the initiation, coordination, and maintenance of the implementation of “Karl-Heinz” in their centers. Additionally, they welcome the patients on their first day, explain the procedures during prehabilitation, and serve as contact persons for questions. Regular online supervision is provided by members of the research team (“study researchers”) to discuss the implementation process.

During the kick-off meeting, managers of the prehabilitation centers and multipliers get an overview of the topic, aims, and design of the study.

**“Train-the-trainer” event for the multipliers**

The multipliers receive information as well as material for the intervention (treatment manual) before and during a one-day training (8 hours) to be able to train the health professionals involved in the prehabilitation centers later on. The modules are explained, and general information on the patients’ situation is given. Moreover, the team of the process evaluation introduces dates, contents, and materials concerning their evaluation.

**On-site training sessions by multipliers**

The multipliers hold on-site training sessions with the involved health professionals in their centers before the first patient arrives. They explain the study as well as the focus of the modules and the overall procedures to the health professionals (such as physiotherapists, occupational therapists, psychologists, social workers, physicians). The multipliers also act as contact persons for the intervention health professionals personnel during the prehabilitation period and are responsible for the training of new health professionals .

**Regular supervision contacts of multipliers with the study researchers**

The study researchers regularly supervise the multipliers in one-hour video supervisions once a month, or, if necessary, more often, to discuss the current implementation experiences, occurring problems and barriers, and possible solutions. All supervising study researchers hold a PhD in a health profession and are familiar with the conditions in rehabilitation centers in Germany.

**5. Who provides**

The multipliers implement and maintain “Karl-Heinz” in the prehabilitation centers. Over the duration of the prehabilitation period, they are financed by the study with a full-time equivalent.

Moreover, other prehabilitation health professionals are part of the intervention team, as they directly provide therapy to patients, i.e., sports and exercise therapy, occupational therapy, and cognitive training. Prehabilitation health professionals receive no incentives or expenditure allowances. However, the prehabilitation center receives a fixed sum per patient from the statutory health insurance, which is also part of the study team.

**6. How**

Different modes of implementation are used as training sessions outside and inside the prehabilitation center, complemented by group video supervision. The intervention itself is directly delivered in therapy sessions, training, and the watching of videos by prehabilitation health professionals.

**7. Where**

Patients are recruited via nine recruiting centers in eight locations across Germany (Goettingen, Hanover, Brunswick, Bad Rothenfelde, Bad Bevensen, Oldenburg, Brandenburg, and Ulm)). After randomization at the patient level, half of the patients (n=211) receive the intervention “Karl-Heinz” in the prehabilitation centers. The eight prehabilitation centers are also located in Germany (Lippoldsberg, Bad Lauterberg, Bad Fallingbostel, Bad Rothenfelde, Bad Bevensen, Oldenburg, Brandenburg, and Ulm).

**8. When and how much**

The individualized prehabilitation program “Karl-Heinz” should be implemented within seven days after inclusion in the study, and the cardiac procedure should take place three to five weeks after inclusion in the study. Each patient in the intervention group has to receive the “Karl-Heinz” intervention for a period of 14 days. A set of minimum required therapies is defined, which must be fulfilled during prehabilitation [15]:

Table 2: Modules of the prehabilitation program “Karl-Heinz”

| **Modules of Karl-Heinz** | **Description of the components** | **Target persons** | **Persons responsible for delivery** | **Location** | **Modes of delivery** | **Frequency, dosage and duration** | **Materials**  **used** |
| --- | --- | --- | --- | --- | --- | --- | --- |
| **Module 1**  Sports and exercise therapy | a) Aerobic endurance training  b) Dynamic strength training and flexibility training  c) Coordination training  d) Breathing therapy | Patients | Physiotherapist, sports scientist/ therapists, qualified health professionals | Prehabilitation center | Not applicable | a) at least 3 times/week, 40-60% Watt max, 65-75% HF max, RPE Scale: 11-14,  45-90 min/week  b) two times/week, muscular strength endurance training 10-15 repetitions, 1-3 sets, 30-50% 1 RM, RPE-Scale: 12-13, at least 60 min/week  c) three times/week, at least 90 min/week  d) three times per week in group setting, at least 90 min/week | Not applicable |
| **Module 2**  Occupational therapy | a) Strengthening activities of daily living  b) Brain performance training in occupational therapy | Patients | Occupational therapists, qualified health professionals | Prehabilitation center | Not applicable | a) 1-2/week, 45 min/session  b) 1-2/week, 45 min/session | Not applicable |
| **Module 3**  Cognitive training | a) Everyday memory strategies  b) Attention training  c) Orientation strategies  d) mediation of procedure and daily routine prior to cardiac intervention/surgery | Patients | Occupational therapists, qualified health professionals | Prehabilitation center | Not applicable | a) 1-3 times/week, 10-15 min/session  b) 2-5 times/week, 10-15 min/session  c) each day twice, 5-10 min/session  d) once, 15-30 min | Not applicable |
| **Module 4**  Psychosocial support | Assessment of psychosocial status, reduction of anxiety, psychoeducation, and development of treatment motivation | Patients | Psychologist, qualified health professionals | Prehabilitation center | Not applicable | 2 sessions, 50 min/session | Not applicable |
| **Module 5**  Disease-specific education | a) Help and psychological support in coping with the disease  b) Implementation of a healthy lifestyle  c) Smoking cessation  d) Dealing with stress and mindfulness  e) Prevention, detection and treatment | Patients | Multipliers, qualified health professionals | Prehabilitation center | Not applicable | Every video once, duration between 5-20 min | Videos |
| **Module 6**  Informative talks with relatives | Informative talks with relatives | Relatives | Psychologists | Prehabilitation center | Not applicable | Once, 50 min | Not applicable |
| **Module 7**  Special hygiene training/nutritional intervention | a) Nutrition  b) Special health training | Patients | a) Nutritional therapist  b) qualified health professionals | Prehabilitation center | Not applicable | a) 1-2 sessions, 30 min/session  b) 1 Video | a) Not applicable  b) Video |

**9. Tailoring**

The responsible physician at the prehabilitation center will adapt the content within the different modules of “Karl-Heinz” to the individual medical situation andneeds of each patient, and will communicate these changes to health professionalst at he prehabilitation center . The categories and numbers of modules are fixed, but adaptations might be necessary to accomodate the physical and psychological capacities of the patient. Further adaptations during the implementation period are possible according to the feedback of the health professionals and patients themselves.

**10. Modifications**

“Karl-Heinz” is intended to be adapted to each patient, and further modifications are not planned. Any modifications will be evaluated by the process evaluation.

**11. How well (planned)**

Fidelity should be maintained as multipliers are trained in detail before the first patient is included and are supervised regularly by the study researchers in group video meetings. In these supervisions, the multipliers report problems occurring during implementation and exchange ideas and experiences.

**12. How well (actual)**

The process evaluation will assess the dose, reach, and fidelity of the implementation of “Karl-Heinz”, any modifications and adaptations made to the modules, and collect the perspectives of the involved stakeholders. The results of the process evaluation will be reported within 18 months after “last patient out” (12-month follow-up completed).
