# Supplementary material for: Mixed-methods process evaluation of the “Karl-Heinz” cardiac prehabilitation program in older patients: protocol for the PRECOVERY trial
Source: Trials. 2026 Mar 18;27:283. doi: 10.1186/s13063-026-09608-4 (PMC13063718; doi:10.1186/s13063-026-09608-4)
Supplement: Supplementary file 2 — Additional file 2. Complete list of recruiting centers, prehabilitation centers, and scientific partners of PRECOVERY. [file 13063_2026_9608_MOESM2_ESM.docx]

## Additional file No. 2

**Complete list of recruiting centers, prehabilitation centers, and scientific partners of PRECOVERY**

|  |  | **Executing entity** | **Representatives** |
| --- | --- | --- | --- |
| **Scientific partners** | University Medical Center Göttingen | Department of Geriatrics | Prof. Dr. Christine von Arnim,  Dr. Stephanie Heinemann,  Dr. Carolin Steinmetz |
|  | University Medical Center Göttingen | Department of Psychosomatic Medicine and Psychotherapy | Prof. Dr. Christoph Hermann-Lingen |
|  | University Medical Center Göttingen | Department of General Practice | Prof. Dr. Eva Hummers  Dr. Christiane Müller |
|  | University Medical Center Göttingen | Department of Medical Statistics | Dr. Thomas Asendorf  Prof. Dr. Tim Mathes |
|  | Geriatric Center Ulm, Ulm |  | Prof. Dr. Michael Denkinger,  Dr. Dhayana Dallmeier |
|  | University Medical Center Hamburg-Eppendorf | Department of Health Economics and Health Services Research | Prof. Dr. Hans-Helmut König |
|  | German Sport University Cologne | Department Preventive and Rehabilitative Sport and Exercise Medicine, Institute of Cardiology and Sports Medicine | Jun.-Prof. Dr. Thomas Schmidt |
| **Recruiting centers** | University Medical Center Göttingen | Department of Cardiovascular and Thoracic Surgery | Prof. Dr. Ingo Kutschka,  Dr. Monika Sadlonova |
|  | University Medical Center Göttingen | Department of Cardiology and Pneumology | Prof. Dr. Gerd Hasenfuß |
|  | Hannover Medical School | Department of Cardiothoracic, Transplantation and Vascular Surgery | PD Dr. Bastian Schmack |
|  | Braunschweig Municipal Hospital | Department of Cardiac, Thoracic and Vascular Surgery | PD Dr. Wolfgang Harringer |
|  | Oldenburg Hospital | Department of Cardiac Surgery | Prof. Dr. Andreas Martens |
|  | Bernau-Brandenburg Heart Center | Immanuel Clinic, department of Cardiovascular Surgery | Prof. Dr. Johannes Maximilian Albes |
|  | Ulm University Medical Center | Department for Thoracic, Cardiac and Vascular Surgery | Prof. Dr. Andreas Liebold |
| **Recruiting- and prehabilitation centers** | Schüchtermann-Schiller'sche Clinic, Bad Rothenfelde |  | Prof. Dr. Nils Reiss |
|  | Heart and Vascular Center Bad Bevensen |  | Prof. Dr. A. Remppis, Dr. Christian Baumbach |
| **Prehabilitation centers** | Clinic and Rehabilitation Center Lippoldsberg |  | Prof. Dr. Gerd Hasenfuß,  Dr. Michael Don |
|  | Kirchberg Clinic, Bad Lauterberg |  | Dr. Ernst Knoglinger |
|  | Rehabilitation Center Oldenburg |  | Dr. Daniela Heidkamp |
|  | Clinic Fallingbostel, Bad Fallingbostel |  | Dr. Susanne Brunner |
|  | ZAR Center for Outpatient Rehabilitation GmbH, Ulm |  | Susann Ernst |
|  | Brandenburg Clinic, Bernau |  | Dr. Martin Schikora |
| **Cooperating partners** | AOK Health Insurance | AOK Lower Saxony | Marcus Matzeder |
|  | AOK Health Insurance | AOK Baden-Württemberg | M.Sc. Christine Kleber-Peukert |
|  | German Heart Foundation |  | Martin Vestweber |
