# Supplementary material for: Mixed-methods process evaluation of the “Karl-Heinz” cardiac prehabilitation program in older patients: protocol for the PRECOVERY trial
Source: Trials. 2026 Mar 18;27:283. doi: 10.1186/s13063-026-09608-4 (PMC13063718; doi:10.1186/s13063-026-09608-4)
Supplement: Supplementary file 3 — Additional file 3. Overview of standardized instruments used for the quantitative part of the process evaluation. [file 13063_2026_9608_MOESM3_ESM.docx]

## Additional file No. 3

**Overview of standardized instruments used for the quantitative part of the process evaluation**

| **Instrument (References)** | **Language version used** (Reference for translated versions) | **Outcome domains and subdomains of the process evaluation** | **Items and scaling formats** |
| --- | --- | --- | --- |
| Hamburg Questionnaire on Hospital Stay [35] | German | Rating of specific aspects regarding the prehabilitation center | Statements (e.g.: “The attending physician seemed distant and impersonal to me.”) to be rated by means of a 5-step rating scale ranging from 1= “no” to 5= “yes” |
|  |  | General rating of key elements of the patients’ stay at a prehabilitation center (satisfaction) | Statements (e.g.: “Overall, I rate the accommodation and meals in this center as...”) to be rated by means of a 5-step rating scale ranging from 1= “very good” to 5= “inadequate” |
|  |  | Patients’ opinions and further recommendations regarding implementation and realization of the intervention | Open responses to various statements  (e.g.: “I have the following suggestions for improvement of the intervention: ...”) |
| Patient Questionnaire of the German Pension Insurance quality assurance programme [36] * | German | Rating of specific aspects regarding the prehabilitation center | Statements (e.g.: “The psychologist was empathetic and understanding.”) to be rated by means of a 5-step rating scale ranging from 1= “no” to 5= “yes” |
|  |  | Rating of specific aspects of the prehabilitation | Questions (e.g.: “How would you rate the scope of services (care, treatment and counseling) during prehabilitation?”) to be answered by means of a 5-step rating scale ranging from 1= “far too few” to 5= “far too many” |
|  |  | Extend of utilization of sessions by the patients (reach) as well as feedback on quality of the sessions | Questions (e.g.: “Did you receive sports and exercise therapy?”) to be answered by “yes” or “no”; if answered “yes”, the quality of the session is rated by means of a 5-step rating scale ranging from 1= “very bad” to 5= “very good” |
|  |  | Description of the general procedures during prehabilitation (dose, reach) | Questions (e.g.: “When did your treatment sessions begin?”) to be answered by means of a 5-step rating scale ranging from 1= “on admission day” to 5= “after the 4^th^ day”;  or questions (e.g.: “How often were sessions canceled during the entire prehabilitation period?”) to be answered by means of a 5-step rating scale ranging from 1= “never” to 5= “more than 3 times” |
|  |  | General rating of the prehabilitation (satisfaction) | Statements (e.g.: “The prehabilitation prepared me well for the operation and the time afterward.”) to be rated by means of a 5-step rating scale ranging from 1= “no” to 5= “yes” |
| *Normalization Measure Development QUES (NoMAD)* [37] | German [38] | Implementation strategies and activities: Implementational work within teams in the prehabilitation centers (multipliers, health professionals): Coherence, cognitive participation, collective action, reflexive monitoring | Statements (e.g. “Health professionals have a shared understanding of the purpose of PRECOVERY.”) to be rated by means of a 5-step rating scale ranging from 1= “absolutely agree” to 5= “absolutely not agree” |

*Some of the questions were removed to shorten the survey, and some questions were slightly adapted to fit the conditions in the prehabilitation centers
