# Supplementary material for: Mixed-methods process evaluation of the “Karl-Heinz” cardiac prehabilitation program in older patients: protocol for the PRECOVERY trial
Source: Trials. 2026 Mar 18;27:283. doi: 10.1186/s13063-026-09608-4 (PMC13063718; doi:10.1186/s13063-026-09608-4)
Supplement: Supplementary file 4 — Additional file 4. “Karl-Heinz”-Diary for patients and health professionals. [file 13063_2026_9608_MOESM4_ESM.docx]

“Karl-Heinz”-
Diary

- For Patients -

**ASSESSMENT OF YOUR CONDITION**

Please give us **daily** assessments of how you felt on that day (preferably in the evening).

**To do this, enter the date and tick the box below the
corresponding face:**


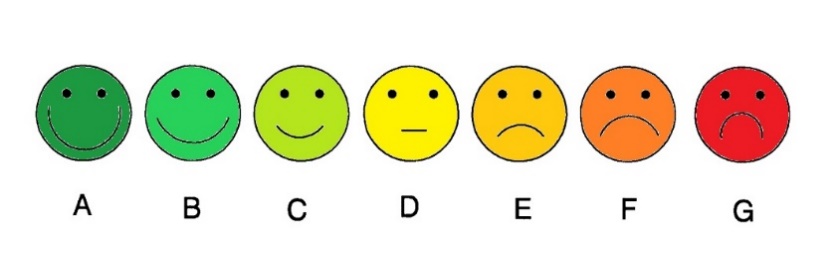


**26 3 2024**

**Example:**
How did you feel overall today, on ___ . ___ . ________?


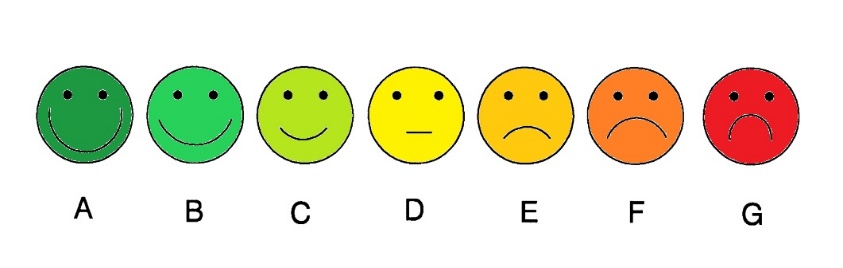


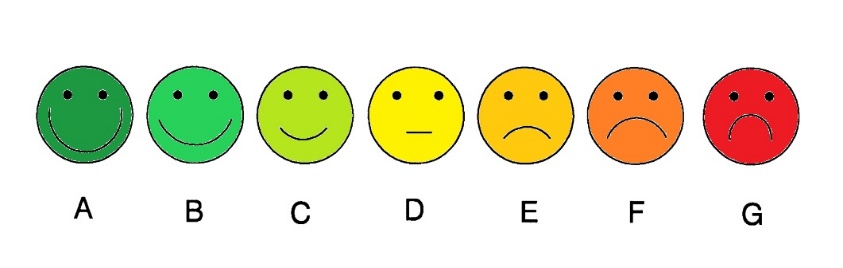

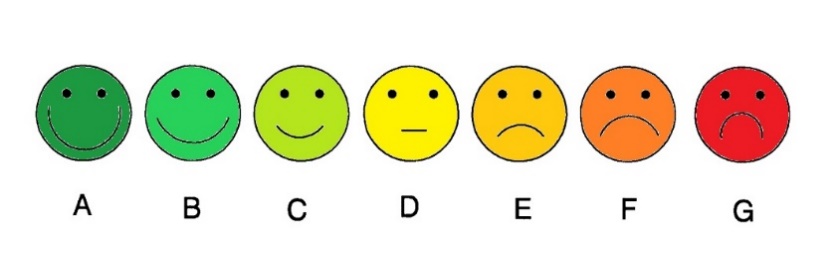


How did you feel overall today, on ___ . ___ . ________?


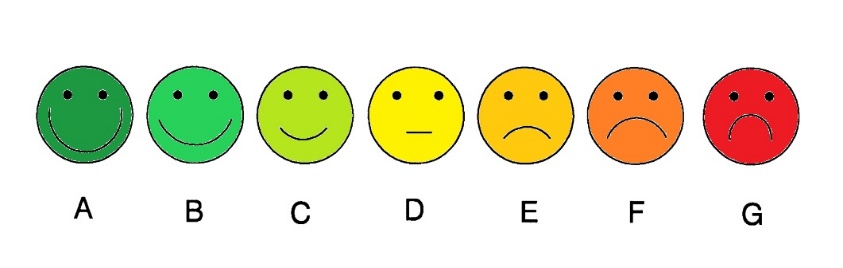

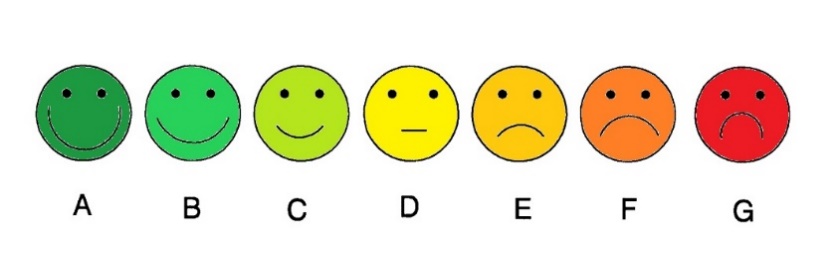


How did you feel overall today, on ___ . ___ . ________?

**X**

**Assessment of the Sessions**

Please **rate the session you just received**.
The numbers refer to the numbering in the white booklet (from page 8).

**Please make a mark on the line:**

**Example:** The session no. 5 was …

**Much too**

**exhausting**

**Much too easy**

**X**

The session no. 1 was …

**Much too**

**exhausting**

**Much too easy**

The session no. 2 was …

**Much too**

**exhausting**

**Much too easy**

The session no. 3 was …

**Much too**

**exhausting**

**Much too easy**

“Karl-Heinz”-
Diary

- For Health Professionals -

Part A

**The content of the session ...**
 was carried out as planned  was adjusted  not applicable

| The session took place on _____. _____ . ________ [date]  from ____:____ to ____:_____ o’ clock. | | |  |
| --- | --- | --- | --- |
| Are you standing in for a colleague?  yes  no | |  |  |
|  | |  |  |
| Module 1: Sports and exercise therapy  Aerobic endurance training  Strength/flexibility training  Coordination training  Respiratory therapy  Module 2: Occupational therapy  ADL (activities of daily living)  Brain performance training  Module 3: Cognitive training  Everyday memory strategies  Attention Training  Orientation strategies  Mediation of procedure / daily routine  Module 4: Psychosocial support   \|  \|  \| \| --- \| --- \| \|  \|  \|  \|  \|   Session 1  Session 2 | **Module 5: Disease-specific education**  Prevention  Coping with the disease  Coping with complications  Implementation of a healthy lifestyle  Smoking cessation  Information about the cardiac  interventions  Mindfulness and dealing with stress  **Other**  Progressive muscle relaxation  Massage  hygiene training/nutrition  The following adjustments have been made: | | |

Gruppentherapie

Einzelbehandlung

“Karl-Heinz”-
Diary

- For Health Professionals -

Part B

**Documentation General Implementation of "Karl-Heinz"**

1. Were the minimum therapy requirements met during the prehabilitation program?  yes  no

If no, please specify:

**Documentation “Informative talks with Relatives”**

1. **Informative talk with relatives**
   1. The talk with relatives has taken place:

yes  no
(If no: please give reasons in the box below)

- 1. Who was spoken to?
     _____________________________
  2. Did anything noteworthy occur during the talk?

If yes, please specify:

yes  no

**Documentation „Module 1: Sports and Exercise Therapy“**

1. **Aerobic endurance training**
   1. Has an exercise ECG been carried out?  yes  no
      1. If yes, what was the maximum power load in watts?
      2. How high did the patient rate the subjective maximum

strain (Borg scale)?

- - 1. Why was the exercise ECG terminated?

| Gen. exhaustion | Fatigue | Motivation |
| --- | --- | --- |
| Leg weakness | Dizziness | Drop in blood  pressure |
| Leg pain | Cold sweat | Angina Pectoris |
| Shortness of  breath | Pallor |  |
| ECG deviations, namely: _____________________ | | |
| Other deviations, namely: __________________ | | |
|  | | |

- 1. How many sessions were completed during the prehabilitation
     program?
  2. How high was the average training load in watts?
  3. What was the average training duration in minutes?
  4. What was the average resting heart rate in beats/min.?
  5. What was the average training heart rate in beats/min.?
  6. What was the average resting heart rate in beats/min.
     (2 minutes after the end of exercise)?

- 1. On average, how high was the training load subjectively
     rated by the patient (Borg scale)?
  2. Did anything noteworthy occur during the training sessions?

yes  no

If yes, please specify:

1. **Strength/flexibility training**
   1. How many sessions were completed during the prehabilitation
      program?
   2. How many strength exercises were performed on average per training session?
   3. Which strength exercises were completed? Tick the appropriate boxes:

| Leg extensions | Leg press | Rowing |
| --- | --- | --- |
| Back extensor | Lat pulldown | Dips |
| Other: _____________________________________________________ | | |

- 1. On average, how high was the training load subjectively rated by the patient (Borg scale)?
  2. Did anything noteworthy occur during the training sessions?

yes  no

If yes, please specify:

1. **Coordination training**
   1. How many sessions were completed during the prehabilitation
      program?
   2. Did anything noteworthy occur during the training sessions?

yes  no

1. **Respiratory therapy**
   1. How many sessions were completed during the prehabilitation
      program?
   2. Did anything noteworthy occur during the training sessions?

yes  no

If yes, please specify:

If yes, please specify:

**Documentation „Module 2: Occupational Therapy“**

1. **ADL (activities of daily living)**
   1. How many sessions were completed during the prehabilitation
      program?
   2. Which content was realized?
   3. Did anything noteworthy occur during the training sessions?

If yes, please specify:

yes  no

1. **Brain performance training**
   1. How many sessions were completed during the prehabilitation
      program?
   2. Which content was realized?
   3. Did anything noteworthy occur during the training sessions?
        yes  no

If yes, please specify:

**Documentation „Module 3: Cognitive Training”**

1. **Everyday memory strategies**
   1. How many sessions were completed during the prehabilitation
      program?
   2. Which content was realized?
   3. Did anything noteworthy occur during the training sessions?
        yes  no

If yes, please specify:

1. **Attention training**
   1. How many sessions were completed during the prehabilitation
      program?
   2. Which content was realized?
   3. Did anything noteworthy occur during the training sessions?
       yes  no

If yes, please specify:

**Documentation „Module 3: Cognitive training”**

1. **Orientation strategies**
   1. How many sessions were completed during the prehabilitation
      program?
   2. Which content was realized?
   3. Did anything noteworthy occur during the training sessions?
       yes  no

If yes, please specify:

1. **Mediation of procedure / daily routine**
   1. How many sessions were completed during the prehabilitation
      program?
   2. Which content was realized?
   3. Did anything noteworthy occur during the training sessions?
       yes  no

If yes, please specify:

**Documentation „Module 5: Disease-specific Education”**

1. **Video education: Prevention, detection, and treatment of
   cardiovascular risk factors and high-risk diseases**
   1. Has the video been watched to the end?  yes  no
   2. Were the questions that arose answered?  yes  no

Comments:

1. **Video education: Help and psychological support in coping with the
   disease**
   1. Has the video been watched to the end?  yes  no
   2. Were the questions that arose answered?  yes  no

Comments:

1. **Video education: Implementation of a healthy lifestyle**
   1. Has the video been watched to the end?  yes  no
   2. Were the questions that arose answered?  yes  no

Comments:

1. **Video education: Smoking cessation**
   1. Has the video been watched to the end?  yes  no
   2. Were the questions that arose answered?  yes  no

Comments:

1. **Video education: Information about major cardiology or cardiac surgery procedures**
   1. Has the video been watched to the end?  yes  no
   2. Were the questions that arose answered?  yes  no

Comments:

1. **Video education: Mindfulness and dealing with stress**
   1. Has the video been watched to the end?  yes  no
   2. Were the questions that arose answered?  yes  no

Comments:

1. **Video education: Recognition and behavior in acute disease-related complications in cardiac surgery or cardiology**
   1. Has the video been watched to the end?  yes  no
   2. Were the questions that arose answered?  yes  no

Comments:
